# The missed potential of CD4 and viral load testing to improve clinical outcomes for people living with HIV in lower-resource settings

**DOI:** 10.1371/journal.pmed.1002820

**Published:** 2019-05-29

**Authors:** Peter D. Ehrenkranz, Solange L. Baptiste, Helen Bygrave, Tom Ellman, Naoko Doi, Anna Grimsrud, Andreas Jahn, Thokozani Kalua, Rose Kolola Nyirenda, Michael O. Odo, Pascale Ondoa, Lara Vojnov, Charles B. Holmes

**Affiliations:** 1 Global Health, Bill and Melinda Gates Foundation, Seattle, Washington, United States of America; 2 International Treatment Preparedness Coalition, Gaborone, Botswana; 3 Médecins Sans Frontières Access Campaign, Geneva, Switzerland; 4 Southern African Medical Unit, Médecins Sans Frontières, Cape Town, South Africa; 5 Clinton Health Access Initiative, Boston, Massachusetts, United States of America; 6 HIV Programmes and Advocacy, International AIDS Society, Geneva, Switzerland; 7 Department of HIV/AIDS, Malawi Ministry of Health, Lilongwe, Malawi; 8 African Society of Laboratory Medicine, Addis Ababa, Ethiopia; 9 World Health Organization, Geneva, Switzerland; 10 Center for Global Health and Quality, Georgetown University, Washington, DC, United States of America; 11 Johns Hopkins University School of Medicine, Baltimore, Maryland, United States of America

## Abstract

In a Policy Forum, Peter Ehrenkranz and colleagues discuss the contribution of CD4 and viral load testing to outcomes for people with HIV in low- and middle-income countries.

**Summary points**

The combination of CD4 and viral load (VL) testing has long been recognized as integral to the management of HIV disease. However, recent shifts in the global HIV response have driven rapid changes in the prioritization and use of these tests.

- CD4 remains the gold standard for assessing disease progression and the need for the World Health Organization’s advanced disease package of screening, prophylaxis, or treatment for major opportunistic infections, which decreases mortality among eligible individuals. Yet, support for site-level provision of CD4 testing capacity is declining.
- Access to routine VL testing, which is the focus of most laboratory resources in low- or middle-income countries (LMICs), is required to assess an individual’s response to treatment. Results should lead to VL-informed differentiated service delivery and better clinical decision-making, including referral to intensified or less frequent clinical care.
- However, available evidence suggests that the majority of CD4 and VL tests currently performed in LMICs do not lead to changes in clinical management, drawing urgent attention to the need to rethink the approach to testing and use of results along with continued efforts to improve access to the tests.
- Frontline access to CD4 and VL remain essential. Yet, a laboratory system is only as good as its ability to return a result to patients and their providers. HIV programs and funders could improve clinical outcomes by measuring the success of their laboratory investments not just by the numbers of tests performed but by the ability of those test results to lead to better outcomes among people living with HIV.

## Introduction

CD4 and viral load (VL) testing have long been recognized as integral to the management of HIV disease [1,2]. However, recent shifts in the global HIV response have driven rapid changes in the prioritization and use of these tests. Perhaps most critical, the World Health Organization (WHO)’s 2016 recommendation to treat all people diagnosed with HIV regardless of immune status led to the loss of one of the primary indications for CD4 testing [1]. In addition, the perceived value and feasibility of VL testing has risen rapidly, driven by decreasing costs of VL testing [3], WHO’s 2013 recommendation encouraging use of VL over CD4 for routine monitoring [4], and by the 2014 Joint United Nations Programme on HIV/AIDS 90–90–90 campaign [5]. The “third 90” promotes the goal of 90% viral suppression among people on treatment, which modeling suggested was critical to reaching the global vision of controlling the HIV epidemic by 2030 [3].

While CD4 testing continues to serve the critical role of identifying individuals with advanced HIV disease and elevated mortality risk [1], the scale-up of VL has absorbed most of the attention and resources dedicated to laboratory services [6,7]. Meanwhile, available data suggest that the majority of CD4 or VL tests performed in low- or middle-income countries (LMICs) do not lead to changes in clinical management, drawing urgent attention to the approach to testing and use of results [8–10].

### The role of CD4 testing has evolved, but it is still essential for management of HIV

CD4 was initially recommended by WHO for use in three settings: 1) as a major criterion for treatment initiation, 2) monitoring for treatment failure, and 3) risk stratification. With each successive set of HIV guidelines, the immunological threshold for treatment initiation rose: from 200 cells/μL in 2002, to 350 cells/ μL in 2009, to 500 cells/ μL in 2013, and ultimately to treatment for all regardless of CD4 cell count in 2016 [1]. The second use case for CD4, monitoring for treatment failure, was discontinued in 2013 when it was found to be inferior to routine VL monitoring [4,11].

WHO’s remaining recommended use for CD4 is risk stratification, both at treatment initiation and for patients re-presenting for care with virological failure and/or clinically advanced disease (Box 1). This is a significant shift from the routine CD4 monitoring once recommended, but it is a still high-profile role that maintains CD4 as a critical test on the path of quality HIV care. Although clinical staging can be a proxy for immune status, it is not an effective one. In a four-country study, nearly 50% of people with CD4 count <100 cells/μL were classified as having WHO clinical stage 1 or 2 disease [12]. CD4 therefore remains the gold standard for assessing disease progression and the need for the more intensive model of care for people with advanced HIV disease defined in the 2017 WHO guidelines, including screening, prophylaxis or treatment for major opportunistic infections (e.g., tuberculosis and cryptococcal meningitis), rapid antiretroviral therapy (ART) initiation, and intensified adherence counseling [13].

**Box 1. Current indications for CD4 testing of people living with HIV**

1. Baseline risk stratification for patients initiating therapy
2. Ad hoc testing of patients with clinical signs of advanced disease (e.g., in clinic and inpatient settings)
3. One-time risk stratification of individuals re-presenting to care after a substantial period of absence (especially those with VL > 1,000 copies/ml)

Despite continuing gains in treatment coverage, CD4-based risk stratification remains essential. Of the approximately 2 million people starting or restarting ART every year [14], as many as 20%–35% of them have a CD4 cell count less than 200 cells/μL and meet the definition of advanced HIV disease [15,16]. These figures are reflected in higher than expected HIV/AIDS mortality rates—annual deaths due to HIV/AIDS have plateaued at around 1 million per year, double the 2020 Fast Track goals [17]. Although some people at highest risk of death may be identified by the diagnosis of an obvious opportunistic infection or awareness of a high VL, many would be missed without a CD4 test [12].

In addition, increasing proportions of individuals on ART incur treatment interruptions, which often lead to decreased CD4 cell counts and unsuppressed VLs [16,18]. The need for “restaging” upon re-presentation is most urgent after long gaps in care and in the inpatient setting, where, in many cases, two-thirds of individuals have been on treatment previously, and mortality risk is substantially elevated [19].

Some declines in CD4 usage are expected as its use becomes more focused on risk stratification, and between 2017 and 2018, the numbers of CD4 tests conducted in LMICs decreased from 19.2 million to 15.7 million [20]. However, despite the clear clinical case for access to CD4 testing, its overall perceived value has declined among funders and, as a result, with LMIC governments [21]. Together, these trends may threaten the existence of CD4 laboratory networks and the timely availability of testing at frontline health sites.

In this environment, some resource-constrained settings may choose to centralize their CD4 testing capacity in referral centers that manage advanced disease, providing access to peripheral centers via sample transport. Alternatively, since the test’s main function is to rapidly identify those at highest risk of morbidity [13], other settings may choose to augment central testing with point-of-care (POC) CD4 devices prequalified by WHO that have demonstrated improved test result return rates, increased retention, and other outcomes [22,23]. Within a few years, a lateral flow assay may further improve access. One company has created an instrument-free assay that may provide a semiquantitative result at a threshold of <200 cells/μL and is currently undergoing clinical testing [24]. Regardless of testing modality, timely use of CD4 test results for identifying those with advanced disease will optimize clinical outcomes.

### Scale-up of VL testing is underway, yet poor use of test results limits patient benefits and value for money

Following WHO’s 2013 recommendation to implement routine VL testing, many LMICs used both domestic and donor resources to accelerate scale-up of VL. In 2017, over 14 million VL tests were conducted in LMICs, and testing volumes in these settings may reach as high as 29 million tests by 2022 [20]. The prices paid per test can vary significantly across countries, buyers, and platforms based on a variety of factors, including the size of orders, the bargaining power of countries and buyers, and the technologies used. The Clinton Health Access Initiative’s recent 5-country analysis of two leading VL technologies found that all-in costs per test ranged between US$14–$22 [20].

Ideally, improved access to routine VL results should lead to better clinical decision-making, including VL-informed differentiated service delivery (DSD). DSD is the concept that HIV care should be adapted to people’s needs in ways that may reduce unnecessary burdens on individuals and the health system and improve outcomes [25,26]. A viral load result allows for 1) referral of individuals with unsuppressed VL to enhanced care, including additional adherence counseling sessions, repeat testing, and, if needed, a change to second-line therapy; and 2) referral of those with suppressed results to (or maintenance in) less intense models of care, such as collection of refills on a less frequent basis or in a nonfacility location. In addition, the result provides opportunities to emphasize the health benefits of adherence both to the individuals and also to their sexual partners: if they can acheive an undetectable viral load, they cannot transmit HIV sexually (i.e., Undetectable = Untransmittable) [27]. If scaled in combination, these three responses to a VL test result are expected to lead to improved individual and population-level clinical outcomes that would justify the investment in VL testing [28,29].

Such promise has led to rapid improvements in access to VL testing in recent years. However, challenges with the use of results for individual management are only starting to garner the attention they require [9,10,30]. For instance, a number of countries, including South Africa, Namibia, Kenya, and Uganda report VL testing among >75% of individuals on ART each year [31–33]. Yet, a recent Kenyan study demonstrated that diminishing proportions of individuals with unsuppressed VLs complete each step of the VL testing cascade, with only 50% of individuals with unsuppressed VL receiving enhanced adherence counseling (EAC), and only 35% receiving a confirmatory/repeat VL test as required in the national guidelines [34]. A quality improvement (QI) intervention has been developed and is currently being implemented in Kenya to respond to these suboptimal results [35]. In Malawi, where access to VL is rapidly expanding (now reaching an estimated 60% of those in need) [36], the government has developed a database to track and encourage use of the results on a national scale. At baseline, as few as one-third of the people with a VL > 1,000 copies/ml who did not resuppress after adherence counseling received the recommended change to second-line ART [37]. Malawi has developed an intensive process to address these low rates of results use, including a push to decentralize decision-making on access to second-line ART and attempts to drive uptake of lower-intensity care models among those with suppressed VLs. These issues remain valid regardless of the threshold of viral detection available in a given country or the frequency with which testing is required.

### The costs of laboratory tests can be better justified by improving their impact on clinical outcomes

The costs of underutilized laboratory tests—CD4, VL, or any other—are substantial. In addition to the direct costs of the instruments and commodities, infrastructure, human resources, and sample transport, there are the anticipated poorer outcomes among patients whose medications were not appropriately managed and the opportunity costs of not spending these resources on another priority. While there are clear programmatic benefits to assessing the proportion of individuals on ART who are virally suppressed (the “third 90”) on a national level, such aggregate data could be more efficiently and perhaps more accurately assessed with routine surveillance at a representative sample of sites or through cross-sectional national studies [38].

Successful scale-up or maintenance of routine individual-level testing requires a systems approach that goes beyond procurement of commodities, expansion of laboratory testing capacity, choice of centralized versus POC testing, or decision about which specimen to collect (e.g., dried blood spot or plasma). First, the placement of platforms within networks and staffing patterns could be managed such that there is high coverage of testing needs and each machine’s capacity is optimized. Second, scale-up requires finding efficiencies, such as service delivery contracts with the machine manufacturers that include training, use of text messages for automated results return to patients, and other novel contractual features in addition to machine rental and commodities. Third, successful scale-up planning could emphasize that laboratory systems be patient-centered, giving as much attention to the preanalytic and postanalytic activities as to the lab testing itself. Furthermore, laboratory testing recommendations are dynamic and require responsive systems. For instance, both the increase in numbers of people living with HIV (PLHIV) leaving and re-entering care as well as the emergence of drugs with higher resistance thresholds such as dolutegravir within both first- and second-line regimens may affect the optimal frequency of, and response to, VL testing [39].

Indeed, a laboratory system is only as good as its ability to return a result to a patient and lead to a change in clinical management. PLHIV and their providers should be armed with the information needed to demand access to testing and insist on the policy and programming changes to ensure that their results are used. Basic requirements are described in Table 1. The QI efforts in Malawi and Kenya have demonstrated the path to addressing these and other key concerns, but spreading and sustaining the lessons will require deliberate attention from national programs, donors, recipients of care, and implementing partners.

**Table 1:** Putting the patient at the center of the laboratory system to facilitate use of the results.

Create demand and improve treatment literacy	Provide PLHIV and their providers the information needed so they can demand access to testing and insist on the policy and programming changes to ensure that their results are appropriately used.
Accelerate return of results	Implement innovative ways of returning VL results to facilities and even directly to individuals when possible (e.g., via short text messages).
Strengthen providers’ ability to use VL for client management	Create streamlined guidance for providers and job aids with clearly stated thresholds for VL failure and recommendations for repeat testing. Ensure that enhanced adherence counseling and explanation of results are conducted with fidelity to best practices.
Strengthen data systems	Develop better electronic or paper data systems for tracking outgoing samples and results longitudinally (e.g., high VL registers).
Expand access to treatment	Improve availability and access to more tolerable second-line drugs and advanced disease intervention packages.
Implement continuous QI	Support continuous QI initiatives that use data to enhance the efficiency and effectiveness of test access, result use, and overall clinic management [40].

**Abbreviations:** PLHIV, people living with HIV; QI, quality improvement; VL, viral load.

### A call to action

Both CD4 and VL are essential tools for management of HIV, but their full impact remains unrealized in most LMICs. The importance of CD4 for identifying and treating people at risk of advanced disease has been displaced by attention to scale-up of VL testing, the results of which remain grossly underutilized. Lifesaving benefits for PLHIV can be achieved by ensuring that CD4 testing remains widely available to frontline providers and is linked directly to an effective advanced disease package proven to decrease HIV-related mortality. Likewise, every VL test performed should lead to improved use of interventions such as less intense models of care or rapid access to second-line treatment.

To maximize the value of and efforts to scale up clinical monitoring tests, HIV programs and donors are encouraged to commit to measuring success not by the numbers of tests performed but by the improved clinical outcomes achieved through the use of test results. People with HIV deserve to know their results, be engaged in their care decisions, and benefit from the use of test results. The United States President’s Emergency Plan for AIDS Relief (PEPFAR) should be lauded for including support for these concepts in its 2019 Country Operational Guidance and its encouragement to countries to monitor change in clinical outcomes through routine data or targeted QI programs [21]. Yet, continuous support for both CD4 and VL testing and the laboratory:clinical interface will be required to bring these interventions and their associated impact to fruition.

## Abbreviations

ART: antiretroviral therapy
DSD: differentiated service delivery
EAC: enhanced adherence counseling
LMIC: low- or middle-income country
PEPFAR: President’s Emergency Plan for AIDS Relief
PLHIV: people living with HIV
POC: point of care
QI: quality improvement
VL: viral load
WHO: World Health Organization
